# Lipid metabolism and antioxidant system contribute to salinity tolerance in halophytic grass seashore paspalum in a tissue-specific manner

**DOI:** 10.1186/s12870-023-04358-w

**Published:** 2023-06-24

**Authors:** Ling Pan, Xu Hu, Li Liao, Tingchen Xu, Quanquan Sun, Minqiang Tang, Zhenbang Chen, Zhiyong Wang

**Affiliations:** 1 Key Laboratory of Genetics and Germplasm Innovation of Tropical Special Forest Trees and Ornamental Plants, Ministry of Education, College of Forestry, Hainan University, 58 Renmin Avenue, , Haikou, 570228 Hainan China; 2 College of Animal Science and Technology, Yangzhou University, 88 South Daxue Road, Yangzhou, 225009 Jiangsu China; 3 College of Tropical Crops, Hainan University, 58 Renmin Avenue, Haikou, 570228 Hainan China; 4 Department of Crop and Soil Sciences, University of Georgia, 1109 Experiment Street, Georgia Station, Griffin, GA 30223 USA

**Keywords:** Seashore Paspalum, Salt Tolerance, Antioxidant system, Amino acid metabolism, Lipid metabolism

## Abstract

Soil salinization is a growing issue that limits agriculture globally. Understanding the mechanism underlying salt tolerance in halophytic grasses can provide new insights into engineering plant salinity tolerance in glycophytic plants. Seashore paspalum (*Paspalum vaginatum* Sw.) is a halophytic turfgrass and genomic model system for salt tolerance research in cereals and other grasses. However, the salt tolerance mechanism of this grass largely unknown. To explore the correlation between Na^+^ accumulation and salt tolerance in different tissues, we utilized two *P. vaginatum* accessions that exhibit contrasting tolerance to salinity. To accomplish this, we employed various analytical techniques including ICP-MS-based ion analysis, lipidomic profiling analysis, enzyme assays, and integrated transcriptomic and metabolomic analysis. Under high salinity, salt-tolerant *P. vaginatum* plants exhibited better growth and Na^+^ uptake compared to salt-sensitive plants. Salt-tolerant plants accumulated heightened Na^+^ accumulation in their roots, leading to increased production of root-sourced H_2_O_2_, which in turn activated the antioxidant systems. In salt-tolerant plants, metabolome profiling revealed tissue-specific metabolic changes, with increased amino acids, phenolic acids, and polyols in roots, and increased amino acids, flavonoids, and alkaloids in leaves. High salinity induced lipidome adaptation in roots, enhancing lipid metabolism in salt-tolerant plants. Moreover, through integrated analysis, the importance of amino acid metabolism in conferring salt tolerance was highlighted. This study significantly enhances our current understanding of salt-tolerant mechanisms in halophyte grass, thereby offering valuable insights for breeding and genetically engineering salt tolerance in glycophytic plants.

**Supplementary Information:**

The online version contains supplementary material available at 10.1186/s12870-023-04358-w.

## Introduction

Salt accumulation in arable soils is a growing concern for global agriculture [1]. High soil salinity can significantly impede plant growth and reduce crop yield, as most crops are highly sensitive to saline conditions [2]. To address this pressing issue, halophytes are increasingly recognized as essential genetic models for studying the molecular mechanisms behind salt tolerance, with potential to develop plant germplasm that can thrive in saline conditions and enable salinized agricultural land use [1].

Halophytes, equipped with various physiological and biochemical mechanisms, can achieve salt tolerance [3]. However, excess Na^+^ from saline soil can lead to osmotic and oxidative stress, resulting in salt-induced changes in metabolic status, membrane remodelling, and gene and protein expression [1, 4]. To prevent toxic Na^+^ accumulation in plant tissues, halophytes efficiently regulate soil Na^+^ uptake [5].

Due to the high energy cost of adaptation, the antioxidant response, also known as oxidative stress tolerance, is of increasing interest for genetic engineering of salt-tolerant crops [6–8], as halophytes have evolved a complex antioxidant system to combat the toxic effects of reactive oxygen species (ROS) induced by salt stress, with hydrogen peroxide (H_2_O_2_) being the most stable form of ROS. ROS-scavenging enzymes, such as superoxide dismutase (SOD), ascorbate peroxidase (APX), and catalase (CAT) [9, 10], as well as non-enzyme antioxidants, including amino acids (e.g., ascorbate, proline, glutathione), flavonoids, polyamines, and polyols, are necessary for exceptional salinity tolerance [11, 12], although the type and quality of ROS players vary among different halophyte species and even within various individual plant tissues [13]. For example, under high salinity conditions, the non-enzymatic antioxidant glutathione was significantly decreased in the halophytic grass *Desmostachya bipinnata* and *Cakile maritima* [14, 15], but significantly increased in halophyte *Salicornia brachiata* [16]. Thus, mechanistic details of the salt-induced antioxidant response in halophyte species remain largely unclear.

Salt stress is a well-known cause of oxidative damage to the biological membrane, and can lead to changes in plant membrane properties [17]. Membrane lipids, which include sphingolipids, sterols, and glycerolipids, play a crucial role in regulating plant membrane fluidity and permeability in response to abiotic stress [18, 19]. Despite some studies showing salt-induced changes in membrane lipid composition in halophytes, the relationship between specific lipid species and the mechanisms underlying salt tolerance remains poorly understood [19–21]. Therefore, further research is necessary to fully explore this correlation.

Seashore paspalum *(Paspalum vaginatum* Sw.), a halophytic turf species, has excellent potential for improving saline soils in tropical and subtropical regions worldwide due to its salt tolerance capacity [22, 23]. However, there is significant genotypic variation in salt tolerance across *P. vaginatum* species [24], underscoring the need to comprehend its molecular basis of salt tolerance. Although the molecular mechanisms underlying its salt tolerance have received significant attention recently [10, 24], our understanding of the mechanisms involved in halophyte tolerance in this grass is still limited. While a recent study suggested that Na^+^ uptake may confer an advantage to seashore paspalum, the correlation between Na^+^ accumulation and salt tolerance remains unclear. Therefore, we compared salt-tolerant and salt-sensitive *P. vaginatum* accessions to elucidate tissue-specific salt tolerance mechanisms based on the antioxidant system, metabolic adaptation, and lipid remodeling. This knowledge has the potential to enhance salinity tolerance of other crops through genetic engineering or selective breeding.

## Materials and methods

### Plant growth conditions and salt treatment

Two contrasting *P. vaginatum* materials, the salt-tolerant accession (UAS 17–18; PI UPG145) and salt-sensitive accession (USA17-26; PI 647910), were previously reported and used for the current study [25]. The materials were acquired from the University of Georgia, and are now being preserved by the Hainan University and the University of Georgia resource nurseries since they have not been deposited in a publicly available herbarium. Stolons from two-month-old *P. vaginatum* plants were hydroponically cultivated in plastic containers (90-mm top diameter × 57-mm diameter width × 135-mm depth) filled with full-strength Hoagland solution (pH = 6.0) in a growth chamber with a 16 h:8 h light: dark at 30/25 ℃, 50%-60% relative humidity and 1000 µmol m^−2^ s^−1^ standard light intensity.

Salt treatments and non-stress treatments were performed on two-week-old plants in the hydroponic plastic containers by supplementing with (and without) 400 mM NaCl. To avoid the undesirable effects of plant salt shock, NaCl concentrations were gradually increased 100 mM each day up to 400 mM NaCl. On the fifth day of salinity stress, the leaves and roots of salt-stressed and non-stressed plants were collected separately. The treatments were each replicated in three hydroponic plastic containers, and six individual plants from each treatment were considered as a biological replicate. Three to six biological replicates were used for the experiments described below. At the beginning and end of the salt stress period, we measured plant weight (in grams), and using the formula (final weight-initial weight) / time period (5 days), we calculated plant growth rate (in grams per day).

### ICP-MS based ion measurement

ICP-MS analysis was used to quantify leaf and root Na^+^ and K^+^ concentrations. Approximately 50 mg of oven-dried samples were ground and dissolved in 2% HNO3, then diluted to prepare five-point calibration standards for Na^+^ and K^+^ concentration. An iCAP Q ICP mass spectrometer from Thermo Fisher Scientific (USA) was used in single KED mode with the following parameters: 1550 W forward power, 14 L/min coolant gas flow, 1.0749 L/min nebulizer gas flow, 0.8 L/min auxiliary gas flow, and 0.02 s dwell time. ST capacity was calculated as ST = (K^+^/Na^+^ in leaves) / (K^+^/Na^+^ in roots) according to Wang [26].

### H_2_O_2_ measurement and antioxidant enzymatic assay

Approximately 100 mg of fresh roots or leaves from three to five biological replicates were used for the following measurements. H_2_O_2_ concentrations were measured using a commercial kit (Product ID: BC3590, Nanjing Jiancheng Bioengineering Institute, Nanjing, China. Superoxide dismutase (SOD) levels including Mn^2+^-SOD activity and Cu/Zn-SOD were measured using a SOD Assay kit (Product ID: A001-2–1; Nanjing Jiancheng Bioengineering Institute, Nanjing, China). Catalase (CAT) levels were measured using a CAT Assay kit (Product ID: BC0205; Beijing, China, Solarbio).

### Lipidomic profiling analysis

Approximately 100 mg of fresh leaves and roots from three biological replicates were frozen into liquid nitrogen, ground to powder and extracted with 300 µL Methanol/1 mL MTBE/300 µL water. LC–MS analysis was conducted using the Nexera UHPLC LC-30A system (Shimadzu, Kyoto, Japan) coupled to a TripleTOF5600 + (AB SCIEX™) mass spectrometer. The column temperature was set at 40 °C and the mobile phase was 0.1% HCOOH-H_2_O (A)-acetonitrile (B) at a flow rate of 0.3 mL/min in gradient elution. ESI source conditions included Ion Source Gas1 (Gas 1) and Ion Source Gas2 (Gas 2) at 50, Curtain Gas (CUR) at 25, and Source Temperature at 500 ℃/450 ℃ (positive ion/negative ion). The Ion Sapary Voltage Floating (ISVF) was set at 5500 V/4400 V (positive ion/negative ion), and TOF MS scan range was 100–1200 Da with a 0.2 s scan accumulation time. The Declustering potential (DP) was ± 60 V. Lipid identification was performed using LipidSearch software 4.0 (Thermo Fisher Scientific, CA, USA).

### Untargeted metabolomics profiling and identification

For metabolite extraction, approximately 100 mg of freeze-dried leaves and roots from three biological replicates were used. These samples were then analyzed through a UPLC-ESI–MS/MS system with the following conditions: UPLC column was a Waters ACQUITY UPLC HSS T3 C18 (1.8 µm, 2.1 mm*100 mm), and the mobile phase consisted of solvent A (pure water with 0.04% acetic acid) and solvent B (acetonitrile with 0.04% acetic acid). A gradient program was used, starting with 95% A and 5% B and gradually shifting to 5% A and 95% B within 10 min. The column oven was set to 40 °C, and the injection volume was 4 μl.

A triple quadrupole linear ion trap mass spectrometer (Q TRAP), API 4500 Q TRAP UPLC / MS / MS system, equipped with an ESI Turbo Ion-Spray interface, operating in positive and negative ion mode, and controlled by Analyst 1.6.3 software (AB Sciex, USA) was used to acquire LIT and triple quadrupole (QQQ) scans. The ESI source parameters were set to: ion source, turbo spray; source temperature 550 ℃; ion spray voltage (IS) 5500 V (positive ion mode) / 4500 V (negative ion mode); ion source gas I [27], gas II (GSII), curtain gas (CUR) were set at 50, 60, and 30.0 psi, respectively; the collision gas (CAD) was high. The instrument was tuned and mass calibrated with 10 μmol/L and 100 μmol/L polypropylene glycol solutions in the QQQ and LIT modes, respectively. The QQQ scans were acquired as MRM experiments with collision gas (nitrogen) set at 5 psi, and individual MRM transitions were optimized for DP and CE.

The SIMCA 14.1 software (Umetrics, Umea, Sweden) was used for multivariate modelling in metabolic profiling, including orthogonal partial least squares discriminant analysis (OPLS-DA) to identify significantly abundant metabolites. Only identified metabolites with a fold change of 2 or ≤ 0.5 and VIP ≥ 1 were considered significant. Pathway enrichment analysis and visualization of metabolomic data were performed using MetaboAnalyst 5.0 and KEGG Mapper (https://www.genome.jp/kegg/tool/map_pathway2.html) [28].

### Transcriptomic profiling and gene expression analysis

Total RNA was extracted using a Plant RNA Kit (Omega Bio-Tek, Norcross, GA, United States) according to the manufacturer’s protocol. Subsequently, cDNA libraries were prepared using the HiSeq 2000 (Illumina Technologies) platform for transcriptome sequencing and de novo assembly. Raw reads were filtered for adapter sequences and low-quality bases with Trimmomatic (v0.32), followed by alignment to the reference genome using HISAT2 (v2.1.0). Gene expression normalization was performed by calculating Reads per Kilobase per Million Mapped Reads (RPKM) based on Mortazavi and colleagues' method. Identification of differentially expressed genes (DEGs) was achieved using the R software DESeq2 package, with a threshold of false discovery rate (FDR) ≤ 0.05 and |log2 FC|≥ 1.5. GO and KEGG pathway analyses were performed to determine DEGs biological functions. The RNA-seq reads were deposited in the NCBI Gene Expression Omnibus (GEO) under GEO accession number: GSE233155, with quality scores evaluated prior to submission.

### Statistical analysis

Statistical significance analysis was calculated using single-factor ANOVA followed by Duncan’s test was performed using GraphPad Prism 8.0 (GraphPad Software Inc., San Diego, CA, USA).

## Results

### In *P. vaginatum*, genotypic variation in salinity tolerance is associated with Na^+^ uptake

**Fig. 1:**
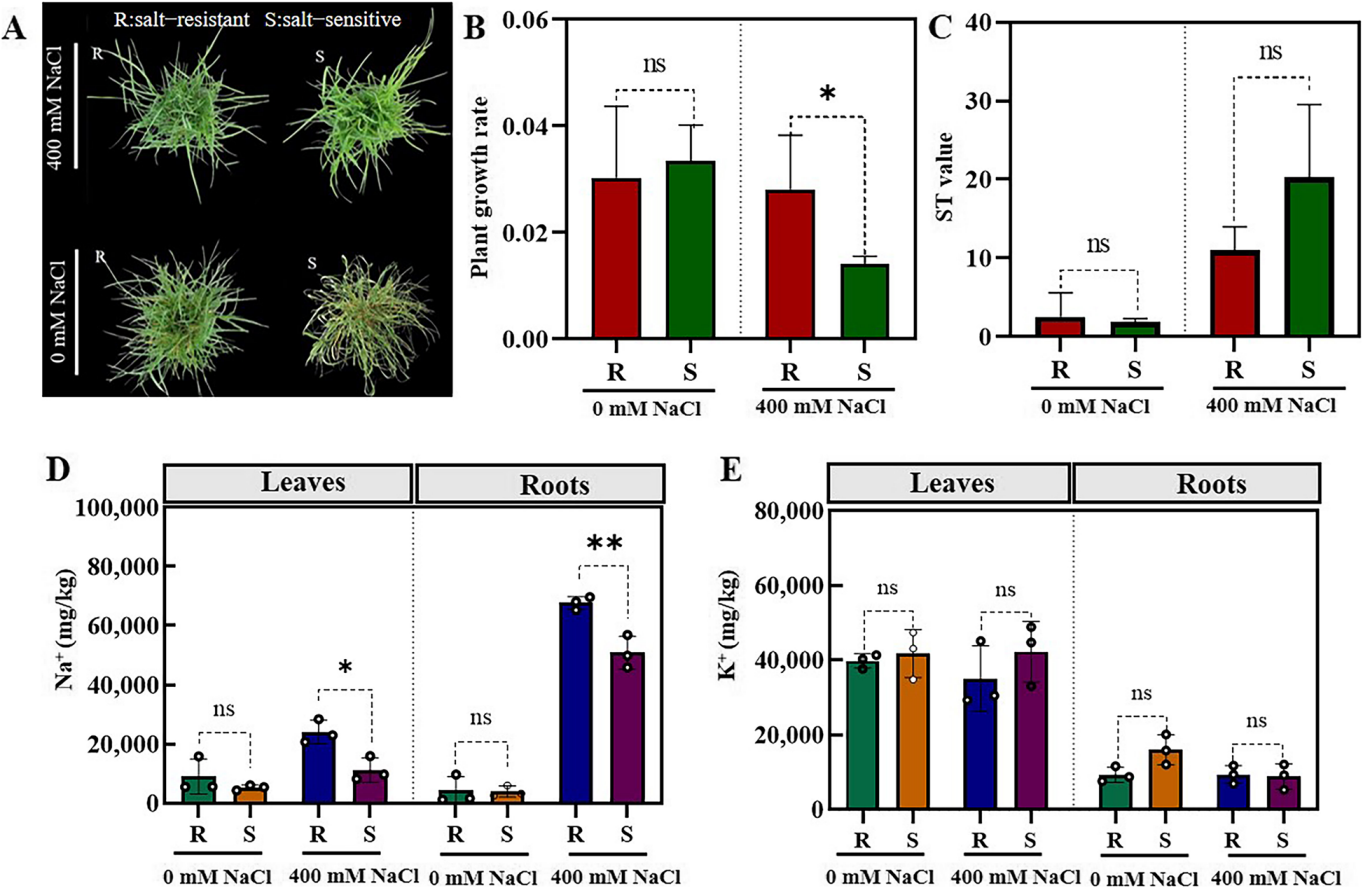
**A** Phenotypical difference of the two *P. vaginatum* accessions treated with high salinity. **B-C** Leaf growth rates and ST values of the two *P. vaginatum* accessions were calculated after 5 days of salt stress. **D-E** Changes in Na^+^ and K.^+^ concentrations in the roots and leaves of salt-tolerant and salt-sensitive plants under salt or non-salted conditions (** significant at *p* < 0.001; ns, not significant; one-way ANOVA followed with two-sided Student’s *t*-test)

After 5 days of NaCl treatment, salt-sensitive plants showed clear symptoms of leaf chlorosis and wilting, while salt-tolerant plants were similar to control plants (Fig. 1A). Notably, a substantial growth rate disparity was observed between salt-sensitive and salt-tolerant plants subjected to 400 mM NaCl treatment (Fig. 1B). In response to high salinity, there was a ssignificant difference in Na^+^ contents of shoots and leaves between salt-sensitive and salt-tolerant plants after NaCl treatment. Both root and shoot Na^+^ increased noticeably in salt-tolerant plants (Fig. 1D). However, K^+^ concentration did not significantly differ between the two accessions under salt stress (Fig. 1E). When exposed to high salinity, Na^+^ ST values did not show significant changes between the two accessions treated with salt stress (Fig. 1C). Our findings suggest that salt-tolerant plants under saline conditions preferentially absorb more Na^+^ and probably store it in *P. vaginatum* roots.

### Na^+^-induced root-sourced H_2_O_2_ activates antioxidant systems in salt-tolerant plants during salt stress

**Fig. 2:**
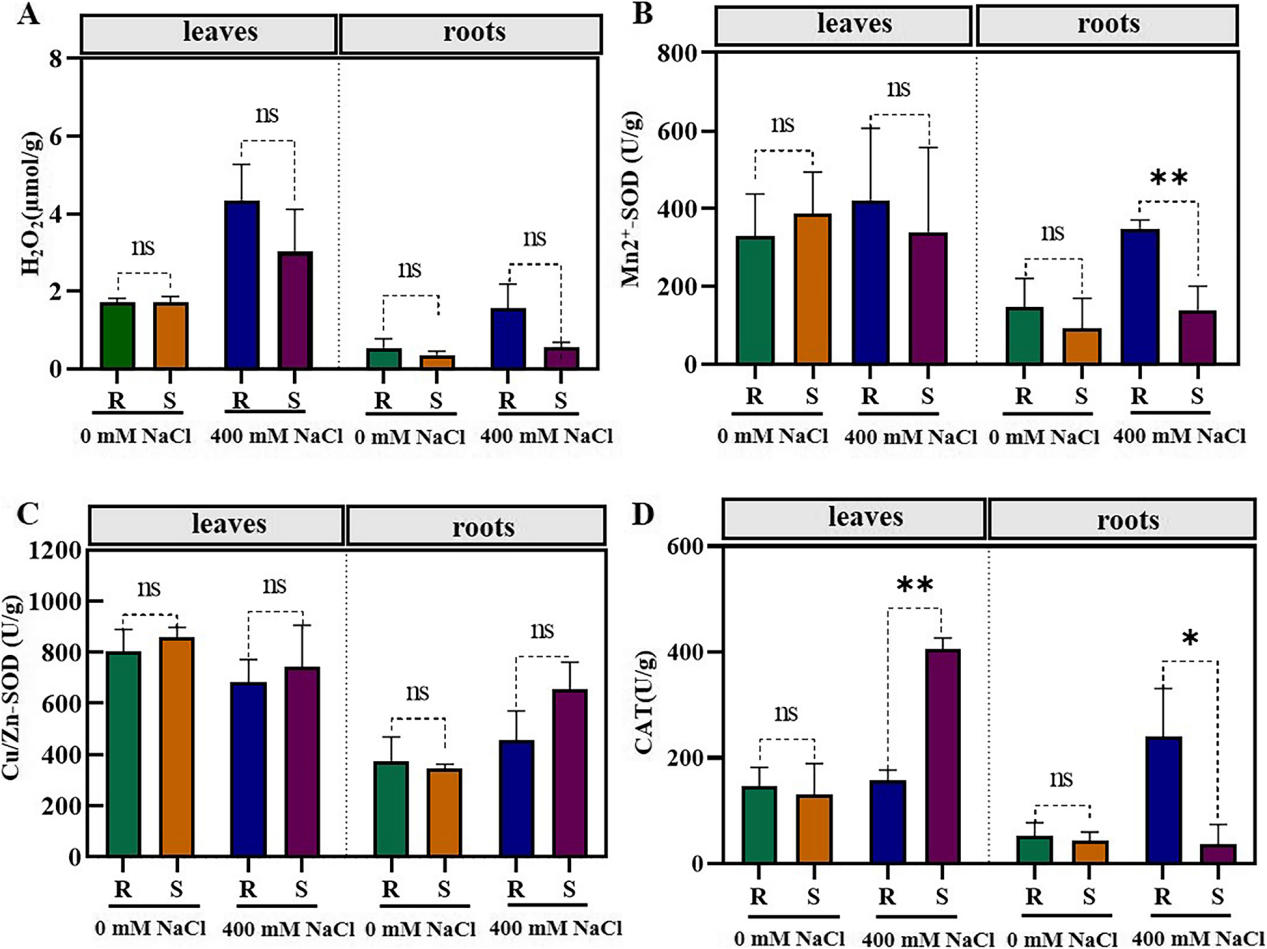
**A-D** Effect of high salinity (400 mM NaCl) on H_2_O_2_ levels and activities of enzymatic players (Mn^2+^-SOD, Cu/Zn-SOD and CAT) in roots and leaves of salt-sensitive and -tolerant plants. (* significant at *p* < 0.05; ** significant at *p* < 0.001; ns, not significant; one-way ANOVA followed with two-sided Student’s *t*-test). Abbrev: ‘R’: salt-tolerant accession; ‘S’: salt-sensitive accession; ‘T’: salt treatment; ‘C’: unsalted treatment; ‘L’: leaf; ‘R.^◦^’: root)

To determine whether such root and shoot Na^+^ accumulation leads to H_2_O_2_ production in salt-tolerant plants after salt treatment, we measured H_2_O_2_ levels and activities of H_2_O_2_-scavenging enzymes in non-salted and treated roots and shoots of the two accessions. A significant increase in Na^+^-induced root-sourced H_2_O_2_ was detected in the tolerant plants, compared with the sensitive plants (Fig. 2A). CAT and Mn^2+^-SOD activities significantly increased in salt-tolerant plant roots, unlike in the salt-sensitive plants (Fig. 2B and 2D). There was no significant accumulation of leaf-derived H_2_O_2_ or Cu/Zn-SOD activity observed in treated leaves following salt treatment, in comparison to either the salt-sensitive plants nor their corresponding controls. (Fig. 2A and 2C). These results suggest that Na^+^-induced H_2_O_2_ is tissue-specific and confers salt tolerance to *P. vaginatum* plants in response to salt stress.

### Metabolome profiling highlights difference between non-enzymatic metabolites from different Na^+^-induced H_2_O_2_

**Fig.3:**
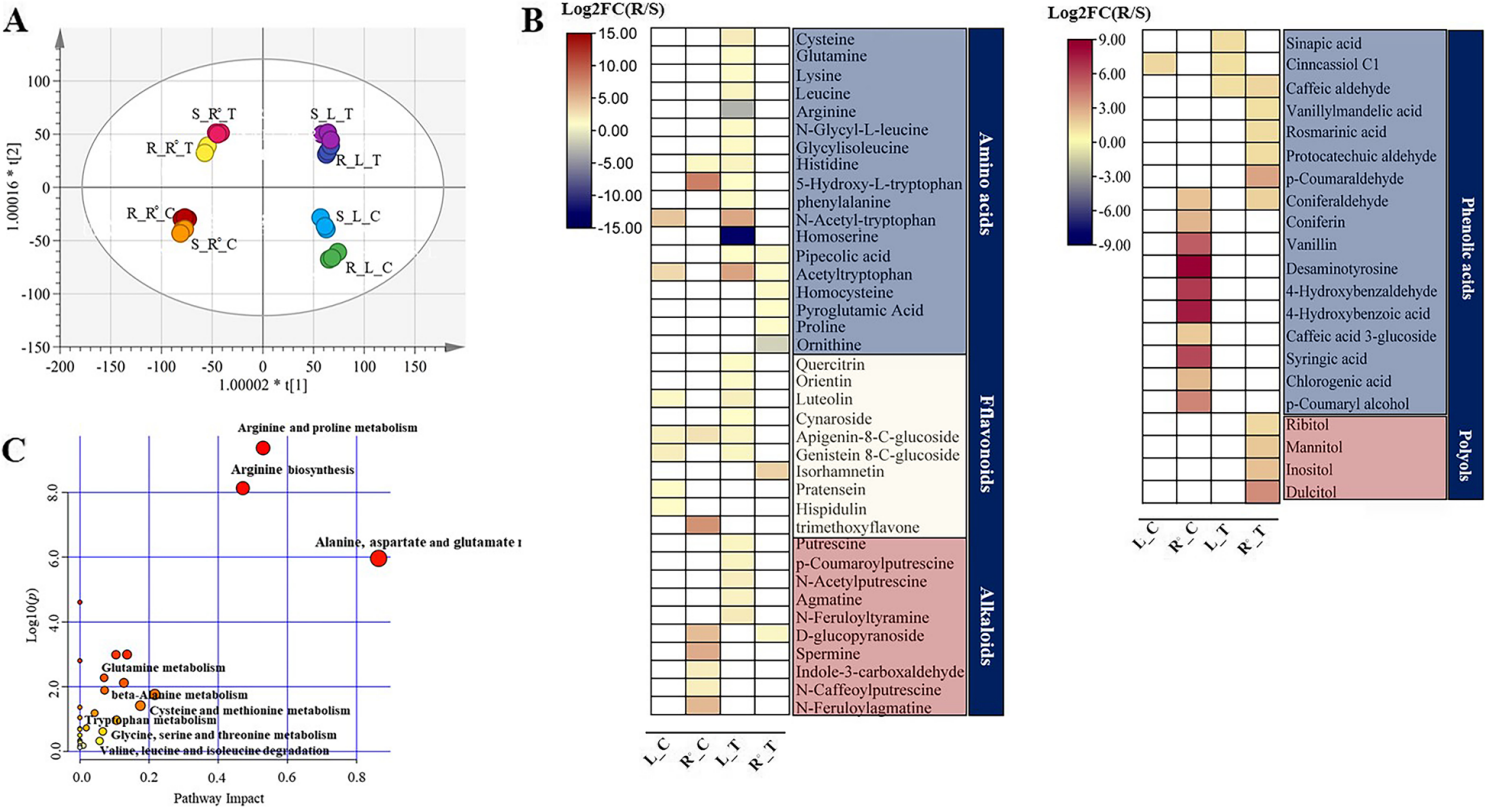
**A** Scatter plots of O2PLS-DA model of different samples for profiling metabolomics. Each symbol represents an independent sample in the score scatter plot and an independent annotated peak in the loading plot; **(B)** Effects of salinity (400 mM NaCl) on metabolic changes in salt-tolerant plants compared to salt-sensitive plants. Heat maps indicate the log2 transferred value of the fold change between salt-tolerant plants and salt-sensitive plants treated with or without 400 mM NaCl. **C** The top enriched KEGG terms of DEGs from leaves in salt-treated tolerant plants of *P. vaginatum* relative to salt-sensitive plants. Abbrev: ‘R’: salt-tolerant accession; ‘S’: salt-sensitive accession; ‘T’: salt treatment; ‘C’: unsalted treatment; ‘L’: leaf; ‘R.^◦^’: root)

We profiled and compared the metabolic status of non-enzymatic antioxidants in the roots and shoots of treated plants of the two accessions (Fig. 3 and Table. S1). After 5 days of NaCl treatment, salt-tolerant plant roots increased abundance of amino acids (such as proline and ornithine), phenolic acids (such as caffeic acid and vanillylmandelic acid) and polyols (such as mannitol and inositol) (Fig. 3C). Instead of phenolic acids and polyols, high salinity resulted in a greater abundance of amino acids (such as proline and pipecolic acid), flavonoids (such as quercitrin, orientin and luteolin), and alkaloids (such as putrescine, agmatine, and spermine) in salt-tolerant plant leaves. These results indicate that Na^+^ induced H_2_O_2_ could confer tissue-dependent metabolic changes in *P. vaginatum* plants.

### High salinity induces lipidome adaptation in P. vaginatum plant roots

**Fig.4:**
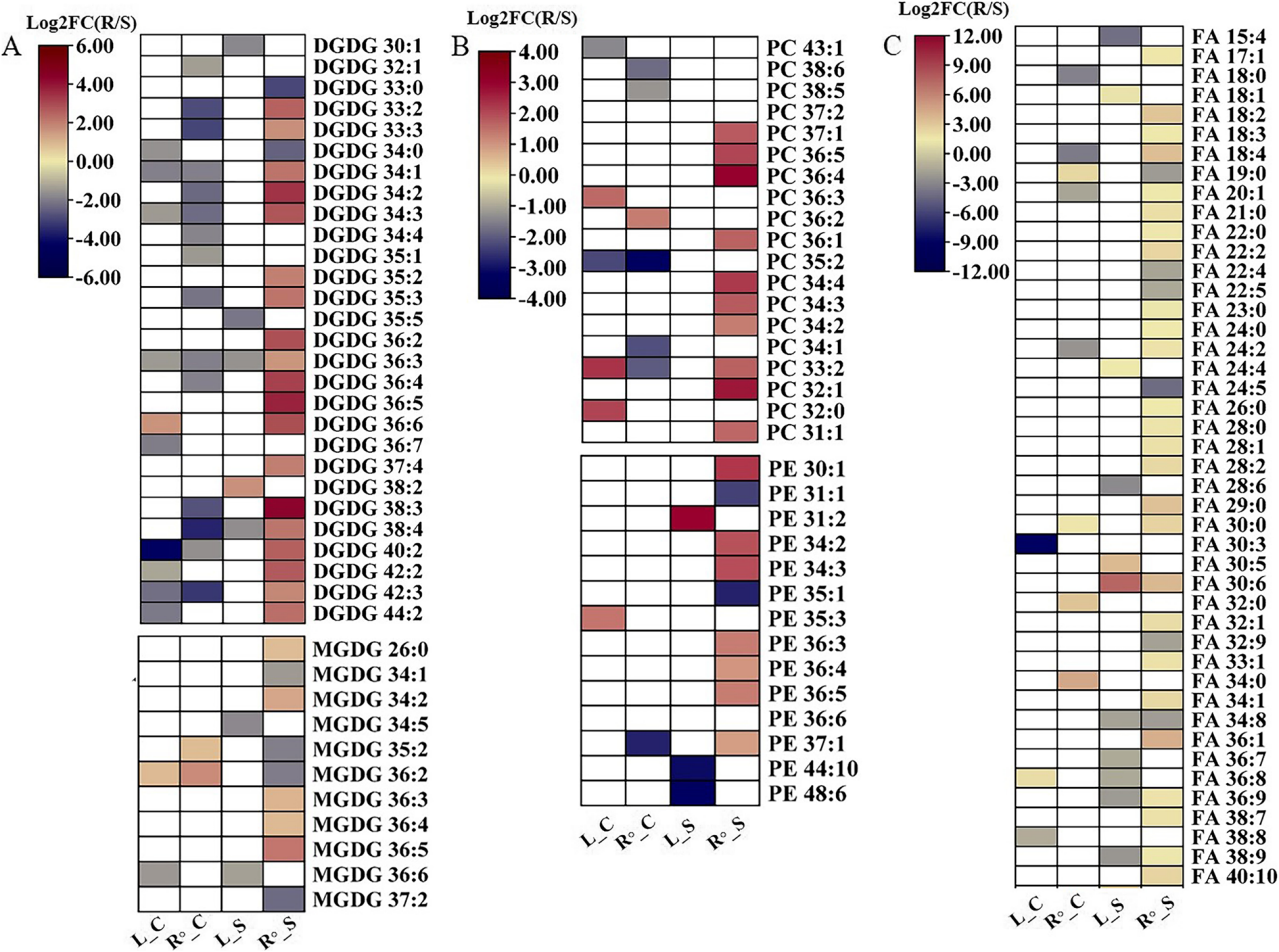
**A-C** Changes in phospholipids, glycolipids and fatty acids in leaves and roots between salt-tolerant and salt-sensitive accessions of *P. vaginatum* under salt stress. Asterisks indicate statistical differences between the indicated treatments, calculated using the Student’s *t-*test with two sides (* significant at *p* < 0.05; ** significant at *p* < 0.001; ns not significant)

To investigate tissue-specific lipid metabolism associated with Na^+^ accumulation in *P. vaginatum* plants, we detected the changes in salt-induced lipid species in both root and shoot between the two accessions*.* Salinity stress elevated root lipidomic species accumulation in salt tolerant plants, including two glycolipids (i.e., MDGD, DGDG and SGDGs), two phospholipids (i.e., PCs and PEs), fatty acids (FA), and triacylglycerols (TGs) (Fig. 4 and Table. S2). Conversely, salt-tolerant plant leaf lipid profiling was not affected by salt stress in *P. vaginatum* plants, as no significant changes in these lipids were observed in leaves of salt-tolerant plants compared to salt-sensitive plants (Fig. 4). These results suggest that Na^+^ accumulation probably triggers upregulation of lipid metabolism to improve *P. vaginatum* salt tolerance.

### Transcriptome profiling highlights salt tolerance-associated genes are inversely related to organic acid and amino acid metabolism

**Fig. 5:**
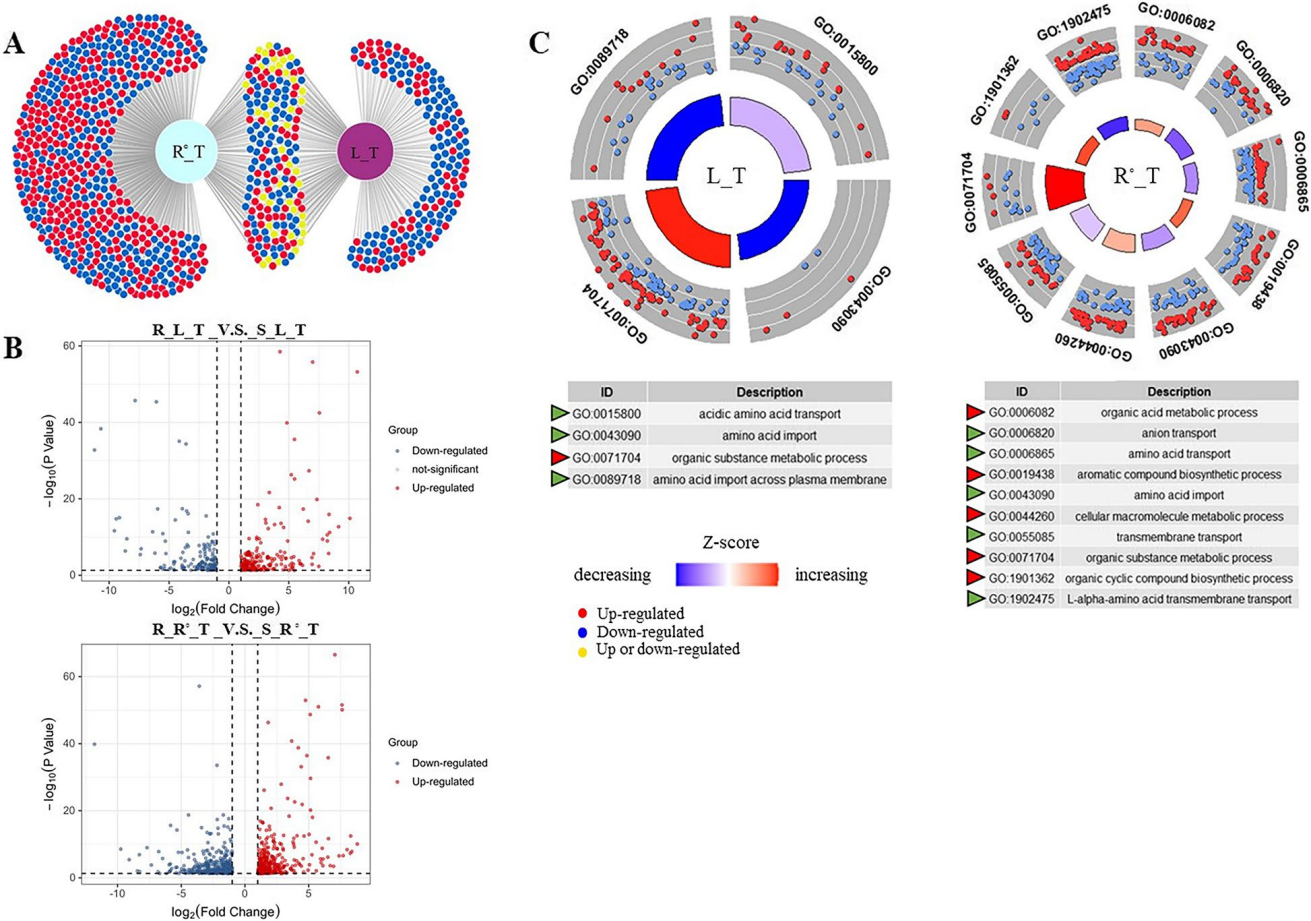
**A** Venn diagram and (**B**) Volcano plot presenting the number and regulation of differentially expressed genes (DEGs) identified from comparative transcriptome analyses of the two *P. vaginatum* accessions under salt stress and non-stress conditions. **C** GO graphs showing the GO terms most enriched involved by DEGs of compound metabolic process in salt-treated leaves and roots. Abbrev: ‘R’: salt-tolerant accession; ‘S’: salt-sensitive accession; ‘T’: salt treatment; ‘C’: unsalted treatment; ‘L’: leaf; ‘R.^◦^’: root)

To explore the key genes associated with salt tolerance in *P. vaginatum* plants, we conducted a comprehensive analysis of differentially expressed genes (DEGs) in the roots and shoots of both *P. vaginatum* accessions under salt stress or non-stress conditions (Fig. 5). In *P. vaginatum* plants, compared to treated leaves, salt-treated roots exhibited a higher number of regulated DEGs (Fig. 5A and Fig. 5B). Gene enrichment analyses revealed that DEGs from salt-treated roots were involved in more biological process pathways related to salt tolerance (Fig. 5C). Under non-stressed conditions, both leaves and roots showed upregulation of DEGs enriched in aromatic and organic compound metabolic processes (Fig. S1). Upon exposure to high salinity stress, DEGs from salt-treated roots were significantly and positively enriched in organic acid metabolic processes and aromatic and organic cyclic biosynthetic processes, while showing a downregulation in amino acid transport (Fig. 5C). Conversely, DEGs identified from leaves were positively related to organic substance metabolic processes in leaves, while negatively enriched in processes related to amino acid import and transport (Fig. 5C).

### Integrated analysis of transcriptome and metabolome highlights amino acid metabolism is essential to plant salt tolerance

**Fig. 6:**
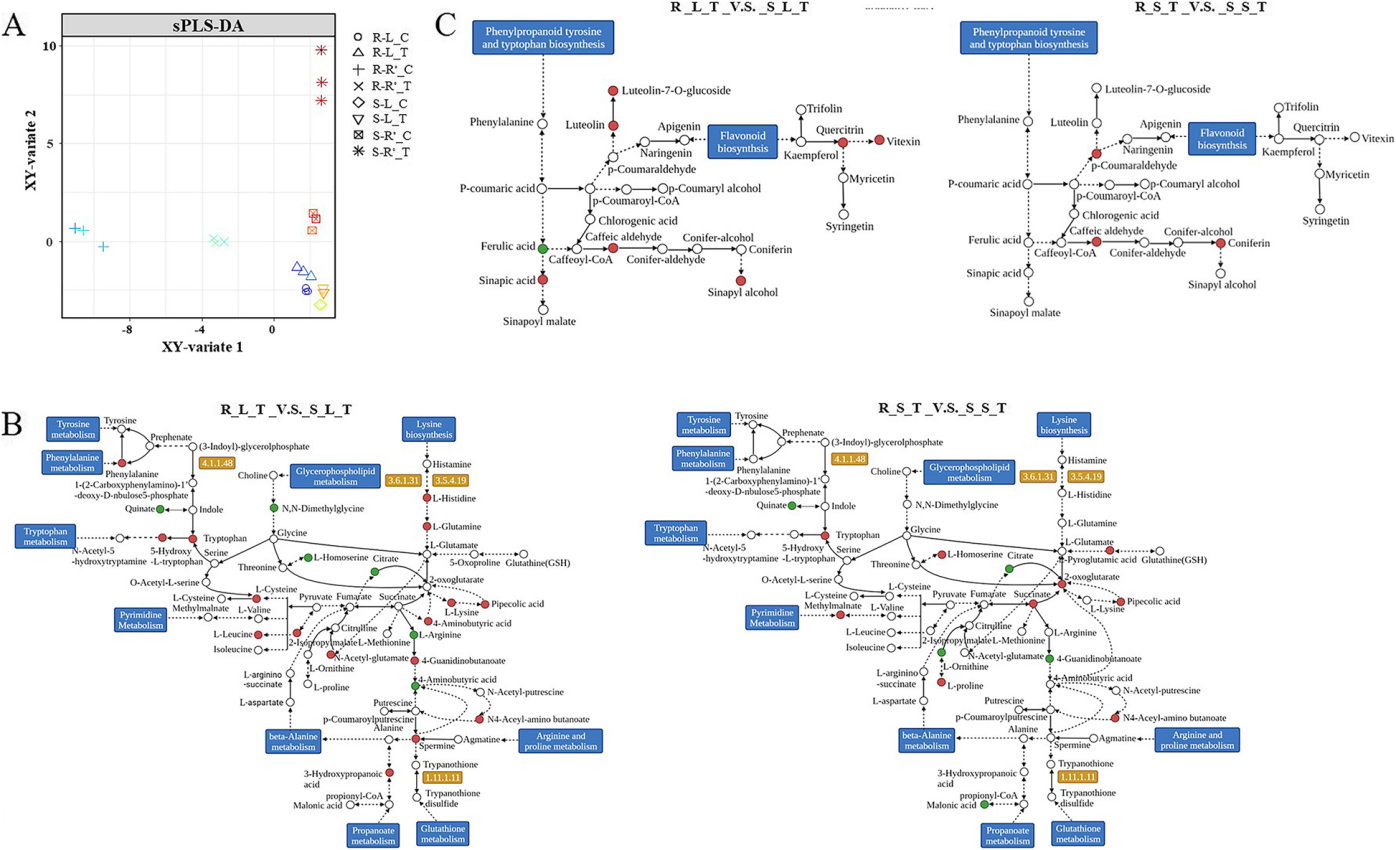
**A** Scatter plots of the sparse PLS-DA model on the first two components using significant metabolites and DEGs based on comparison analysis. **B-C** Complex dynamic regulatory networks of important metabolites involved in amino acid metabolism and their corresponding genes in roots of salt-tolerant *P. vaginatum* accession (The KEGG figures produced under the permission from Kanehisa Laboratories). Solid red or green circles represent either increased or decreased changes in metabolite abundance and gene expression. Abbrev: ‘R’: salt-tolerant accession; ‘S’: salt-sensitive accession; ‘T’: salt treatment; ‘C’: unsalted treatment; ‘L’: leaf; ‘R.^◦^’: root)

Initially, the correlation between important metabolites and their corresponding DEGs on the gene-metabolite regulatory network was assessed using the Partial Least Squares (PLS) regression method (Fig. 6A). Leaf and root individual metabolomes and transcriptomes were well separated based on the sPLS-DA model. An integrated pathway-level analysis was performed to combine the most significant metabolites with their corresponding DEGs in the metabolic networks. Under salt stress in salt-tolerant plants, activation of most metabolic pathways associated with amino acid metabolism played an important switching role in phenolic acids and flavonoids biosynthesis (Fig. 6B and 6C). These findings suggest that during salt stress, amino acids in the shoot may be induced for conversion to phenolic acids and flavonoids for transfer to the roots.

## Discussion

### Na^+^ is required for maintaining salt tolerance in seashore Paspalum

Globally, soil salinization is a growing challenge limiting agriculture. Improved knowledge of salt-tolerant mechanisms can be applied to the development and use of salt-tolerant crop plants. Plants that can tolerate repeated exposure to seawater are considered quite rare. The Seashore Paspalum (*Paspalum vaginatum* Sw.) is one of such ‘rare’ plants. Nevertheless, the precise mechanisms by it deals with high salt concentrations have remained unclear until now.

Recently, Wu et al*.,* (2020) found that higher salinity tolerance of a *P.vaginatum* cultivar was associated with higher Na^+^, which triggered our interest in understanding how Na^+^ accumulation aids its enhanced tolerance[10]. Our study highlighted tissue-specific adaptations as perhaps common among halophytes. High ST values are not necessarily related to a high salt tolerance in a few halophytes such as *Puccinellia tenuiflora* [29], and our results suggest that *P.vaginatum* might be one of those.

As active Na^+^ uptake is efficient and cheaper than osmolyte biosynthesis, it is the principal strategy of some halophytes for osmotic homeostasis under saline conditions [30]. This enables halophytes to maintain cellular turgor pressure and sustain growth even in saline environments. Salt-tolerant *P. vaginatum* accession tends to accumulate Na^+^ to maintain high osmotic pressure for water leaf adsorption [10, 31]. With a combination of physiological and omics evidence, we found that the Na^+^ uptake contributes to osmotic adjustment, lipid adaptation and signalling processes, thereby enhancing salt tolerance in *P. vaginatum* plants.

### Na^+^ accumulation induces root-sourced H_2_O_2_ and subsequently activates the antioxidant system

Extensive studies have shown that hydrogen peroxide (H_2_O_2_) can lead to harmful effects in unfavourable environmental conditions [32, 33]. Nevertheless, several reports highlighted the involvement of root-sourced NADPH-mediated H_2_O_2_ in increasing plant salt tolerance [34–37]. In *P. vaginatum* plants, Na^+^ uptake caused the accumulation of root-organised H_2_O_2_ in salt-tolerant plant roots (Fig. 2A). Our results confirm for the first time that root-sourced H_2_O_2_ plays a vital role in halophyte plant tolerance to high salinity.

SOD and CAT are essential defense mechanisms against oxidative stress caused by salt stress [38]. These two enzymes have been identified as crucial regulators for achieving optimal H_2_O_2_ levels, contributing to plant salinity tolerance in glycophytic plants [39, 40]. However, whether H_2_O_2_ is important for halophytes remains debatable, as there is limited evidence of its importance so far. In our study, increased root-derived H_2_O_2_ did trigger higher amounts of CAT and Mn^2+^-SOD (Fig. 2B and 2D) enzymes suggesting that it induced their activity in roots, therefore contributing to the enzymatic antioxidant system of salt-tolerant seashore paspalum in response to salt stress.

With increasing H_2_O_2_, some amino acids that are important as efficient antioxidants for plants to control the appropriate amounts of H_2_O_2_ under saline conditions were not significantly abundant (Fig. 3B), such as tryptophan, phenylalanine, and phenylpropanoids [41]. Interestingly, we observed that such amino acids were involved in conferring salt tolerance to *P. vaginatum* leaves (Fig. 3B). This result is consistent with the observation that Na^+^ accumulation triggers a slight rise in H_2_O_2_ generation although it was not significantly different between the two accessions (Fig. 1B and Fig. 2A). Our evidence supports that Na^+^ accumulation plays an important role in triggering the enzymatic and non-enzymatic antioxidant system to obtain salinity tolerance in this halophytic species, especially for roots.

### Na^+^ uptake is associated with tissue-specific lipid metabolism

Na^+^ accumulation is commonly thought to decrease lipid contents [24] through lipid peroxidation caused by Na^+^-induced ROS in glycophytic plants under salt stress [42, 43], while studies have provided evidence that membrane lipid alterations are essential for maintaining membrane homeostasis in halophytes under salt stress [19, 20].

In agreement with previous reports, we found that salt-tolerant *P. vaginatum* plants are prone to increase their lipid contents to improve salt tolerance (Fig. 4). The increase was particularly noticeable for galactolipids (DGDGs) and phospholipids (PCs and PEs), and their overall increase has been linked to membrane fluidity and permeability maintenance during osmotic stress [44]. Furthermore, the importance of increased DGDGs, PCs, and PEs contents to plant stress tolerance has been confirmed [24, 45]. We observed an obvious upregulation of DGDGs, PCs and PEs in salt-tolerant plants in response to salt stress, indicating that Na^+^-induced lipid metabolism is required for improved plant tolerance to salt stress in the halophytic grass *P. vaginatum* (Fig. 4).

### Na^+^-induced upregulation of amino acid metabolism is the central hub for root-shoot coordination

As mentioned, several amino acids act as antioxidants in the homeostasis of root-dependent H_2_O_2_ in salt tolerant accession (Fig. 3B). Additionally, some amino acids, such as arginine, are essential precursor molecules for the synthesis of secondary metabolites, including polyamines, phenolic compounds, and alkaloids associated with salt tolerance [46]. For example, the strongly decreased arginine could contribute to polyamines synthesis during salt stress in the leaves of salt-tolerant *P. vaginatum* plants (Fig. 3B), and phenylalanine can serve as precursors for phenolic compounds and alkaloids synthesis to minimise salt stress-induced oxidative stress [47, 48]. This result is consistent with our transcriptome profiling study, in which the gene-metabolite regulatory network has an essential role for amino acids in important secondary metabolites synthesis. Additionally, we identified several DEGs in leaves mapped into GO terms called ‘Amino acid transport” and “Amino acid import’, indicating that amino acids produced in leaves may act as important contributors for polyamines and phenolic compounds required for root salt tolerance.

## Conclusion

Overall, variation in salinity tolerance among different *P. vaginatum* genotypes was closely linked to Na^+^ accumulation in the roots. Under saline conditions, salt-tolerant plants exhibit preferential Na^+^uptake, which is likely stored in their roots. During salt stress, the presence of Na^+^-induced root-sourced H_2_O_2_ activates antioxidant systems in salt-tolerant plants. This activation is tissue-specific and plays a crucial role in conferring salt tolerance. Furthermore, Na^+^ accumulation likely serves as a trigger for the upregulation of lipid metabolism, thereby enhancing *P. vaginatum* salt tolerance. Additionally, DEGs related to salt tolerance are inversely related to organic acid and amino acid metabolism. Overall, this study provides insights into the molecular mechanisms underlying *P. vaginatum* salt tolerance.

## Supplementary Information

**Additional file 1: Table S1.** A Lists of raw data of root metabolomes. **Table S1.** B Lists of raw data of leaf metabolomes. **Table S1.** C Lists of differentially abundanced metabolites from root metabolomes. **Table S1.** D Lists of differentially abundanced metabolites from leaf metabolomes.

**Additional file 2: Table S2.** A Lists of raw data of root lipidomes under different treatments. **Table S2.** B Lists of differential lipids in roots with or without salt stress (p-value<0.05).

## Data Availability

The datasets generated and analyzed for this work were deposited in the NCBI Gene Expression Omnibus under the GEO accession number: GSE233155, available from https://www.ncbi.nlm.nih.gov/geo/query/acc.cgi?acc=GSE233155. Other datasets used in this study are included in this published article and its supplementary information files.
